# Hypotension and Adverse Outcomes in Moderate to Severe Traumatic Brain Injury: A Systematic Review and Meta-Analysis

**DOI:** 10.1001/jamanetworkopen.2024.44465

**Published:** 2024-11-11

**Authors:** Jun Won Lee, Wendy Wang, Amal Rezk, Ayman Mohammed, Kyle Macabudbud, Marina Englesakis, Abhijit Lele, Frederick A. Zeiler, Tumul Chowdhury

**Affiliations:** 1 College of Medicine, University of Saskatchewan, Saskatoon, Saskatchewan, Canada; 2 Temerty Faculty of Medicine, University of Toronto, Toronto, Ontario, Canada; 3 Department of Anesthesiology and Pain Medicine, Toronto Western Hospital, Toronto, Ontario, Canada; 4 Krembil Brain Institute, University Health Network, University of Toronto, Toronto, Ontario, Canada; 5 Library and Information Services, University of Toronto, Toronto, Ontario, Canada; 6 Department of Anesthesiology and Pain Medicine, Harborview Medical Center, Seattle, Washington; 7 Department of Surgery, Rady Faculty of Health Sciences, Winnipeg, Manitoba, Canada

## Abstract

**Importance:**

Traumatic brain injury (TBI) is a leading cause of death and disability worldwide. Hypotension in patients with TBI is associated with poorer outcomes. A comprehensive review examining adverse outcomes of hypotension in patients with TBI is needed.

**Objective:**

To investigate the mortality and incidence of hypotension in patients with TBI.

**Data Sources:**

A search of studies published before April 2024 was conducted using MEDLINE, MEDLINE In Process, ePubs, Embase, Classic+Embase, Cochrane Central Register of Controlled Trials, and Cochrane Database of Systematic Reviews for primary research articles in English, including randomized control trials, quasirandomized studies, prospective cohorts, retrospective studies, longitudinal studies, and cross-sectional surveys.

**Study Selection:**

Inclusion criteria were patients aged at least 10 years with moderate to severe TBI with hypotension. The exclusion criteria were mild TBI (due to the differences in management principles from moderate to severe TBI). Data were screened using Covidence software with multiple reviewers.

**Data Extraction and Synthesis:**

This meta-analysis conforms to Preferred Reporting Items for Systematic Reviews and Meta-analyses (PRISMA) and Meta-analysis of Observational Studies in Epidemiology (MOOSE) reporting guidelines for assessing data quality and validity. Primary outcomes (unadjusted and adjusted odds ratios [ORs]) were calculated using a random-effect model with 95% CIs. Incidence of hypotension was derived using logit transformation.

**Main Outcomes and Measures:**

Main outcomes were association of hypotension with death and/or vegetative state within 6 months and incidence of hypotension. Vegetative state was not reported due to lack of data from included studies. Hypothesis testing occurred before data collection.

**Results:**

The search strategy identified 17 676 unique articles. The final review included 51 studies (384 329 patients). Pooled analysis of found a significant increase in mortality in patients with hypotension and moderate to severe TBI (crude OR, 3.82; 95% CI, 3.04-4.81; *P* < .001; *I*^2^ = 96.98%; adjusted OR, 2.22; 95% CI, 1.96-2.51; *P* < .001; *I*^2^ = 92.21%). The overall hypotension incidence was 18% (95% CI, 12%-26%) (*P* < .001; *I*^2^ = 99.84%).

**Conclusions and Relevance:**

This meta-analysis of nearly 400 000 patients with TBI found a significant association of greater than 2-fold odds of mortality in patients with hypotension and TBI. This comprehensive analysis can guide future management recommendations, specifically with respect to blood pressure threshold management to reduce deaths when treating patients with TBI.

## Key Points

**Question:**

Is hypotension associated with adverse outcomes in patients with moderate to severe traumatic brain injury (TBI)?

**Findings:**

This systematic review and meta-analysis of 51 studies and 384 329 patients found that patients with TBI and hypotension had significantly increased odds of mortality. Incidence of hypotension was 18%.

**Meaning:**

These findings highlight the importance of blood pressure management in TBI settings.

## Teaser

This systematic review and meta-analysis assesses the incidence of hypotension in patients with moderate to severe traumatic brain injury and its association with mortality.

## Introduction

Traumatic brain injury (TBI) is a leading cause of death and disability, with a high incidence of 60 million events per year and 8.1 million years lived with a disability worldwide.^1^ Hypotension is a common secondary injury that occurs in patients with TBI. The etiology of hypotension is multifactorial, commonly caused by excessive blood loss from various injuries. Additionally, previous human studies and research on animal models have proposed neurogenic causes of hypotension. The proposed pathological mechanisms include myocardial depression subsequent to a massive catecholamine release or herniated brainstem disrupting the central autonomic network nuclei and may also involve damage to medullary and hypothalamic structures, which regulate blood pressure (BP).^2,3,4^ Hypotension has been established as a significant contributor to adverse outcomes, including mortality, disability, poor neurological function, and longer intensive care unit stay. Indeed, the current criterion standard prognostic model for moderate and severe TBI, the International Mission for Prognosis and Analysis of Clinical Trials score, includes hypotension (systolic BP [SBP] <90 mm Hg) as one of its determining variables.^5^

Several studies have found that patients with TBI and SBP lower than 90 mm Hg in the prehospital setting experienced poorer health outcomes, such as increased mortality and long-term disability.^6,7^ Aligning with this mounting evidence, a widely used management guideline from the Brain Trauma Foundation recommends avoiding an SBP lower than 100 mm Hg for patients with TBI aged 50 to 69 years and lower than 110 mm Hg for patients with TBI aged younger than 15 or older than 70 years to prevent adverse patient outcomes.^8^ Although there is a collection of literature that focuses on BP and TBI management, the current body of literature lacks systematic reviews and meta-analyses of hypotension in TBI and its associated outcomes. Currently, BP threshold recommendations in the fourth edition of the Brain Trauma Foundation guidelines for TBI management are a weaker level 3 recommendation, due to insufficient evidence. As the global burden of TBI increases, there is an urgent need for more comprehensive systematic reviews and meta-analyses to provide further clinical support and emphasize the importance of managing SBP thresholds in TBI management.

This systematic review aims to present pooled results of available literature and provide a comprehensive up-to-date insight into the association of hypotension with adverse outcomes in patients with TBI. Furthermore, this study will seek to elucidate the association between SBP thresholds and mortality in a more empirical manner in patients with TBI through meta-analysis, while also examining other important variables when assessing patients with TBI with hypotension. Subsequently, this systematic review and meta-analysis can be used to provide evidence to further support and strengthen current guidelines and ultimately help optimize TBI management and treatment.

## Methods

This systematic review and meta-analysis is reported following the Preferred Reporting Items for Systematic Reviews and Meta-analyses (PRISMA) reporting guideline and the Meta-analysis of Observational Studies in Epidemiology (MOOSE) reporting guideline. The protocol for this study was published in the PROSPERO International Prospective Register of Systematic Reviews (CRD42023427560).

### Search Strategy

The comprehensive search strategy was generated with the assistance of an information specialist (M.E.), who generated the list of citations using searches in MEDLINE, MEDLINE In Process, ePubs, Embase, Classic+Embase, Cochrane Central Register of Controlled Trials, and Cochrane Database of Systematic Reviews using the Ovid platform. Searches were conducted in the English language, with parameters to include human-only studies and studies from inception to April 4, 2024. Full details regarding the search strategy are provided in eTable 1 in Supplement 1.

### Inclusion and Exclusion Criteria

The inclusion criteria for studies were patients aged at least 10 years with any form of moderate to severe TBI (initial pilot search yielded many studies with patients within this age demographic, and Pediatric Advanced Life Support guidelines define hypotension for pediatric patients aged ≥10 years similarly as the adult population [SBP <90 mm Hg]^9^), patients exposed to hypotension (as defined in the eAppendix in Supplement 1), and primary research articles in English, including randomized clinical trials, quasirandomized studies, prospective cohorts, retrospective studies, longitudinal studies, and cross-sectional surveys. The exclusion criteria were mild TBI (due to the differences in management principles from moderate to severe TBI); non–English language articles; animal studies; duplicate reports and secondary research articles; abstracts, case series, case reports, reviews, or gray literature; preexisting hypotension in patients prior to TBI; and articles in which the role of hypotension cannot be delineated solely (ie, combined factors, such as hypoxemia or other physiological variables).

### Outcomes

The primary outcome was adverse outcomes following hypotension in patients with TBI. Adverse outcome was defined as a presence of composite Glasgow Outcome Scale–Extended (GOSE) score of 1 (mortality of any type and all cause) and/or 2 (vegetative state) within 6 months.^10^ The main secondary outcome was to note the incidence of hypotension, measured as proportion of patients with hypotension. Both outcomes were also presented based on subgroups (age, BP threshold, hypoxia, TBI scale, TBI severity, multiple-trauma TBI vs isolated TBI, and BP measurement setting). These variables are defined in eTable 2 in Supplement 1. Other outcomes were to assess the duration of hypotension, length of hospital or intensive care unit stay, and management strategies.

### Study Selection

Title and abstract and subsequent full-text screening were carried out using the Covidence software. Independent reviewers (J.W.L., W.W., A.R., A.M., and K.M.) conducted the screening process. Studies were moved to the subsequent level of screening (title and abstract, full-text review, extraction) if 2 authors independently chose to include each respective study during that stage of screening. Disagreements were resolved by the principal investigator (T.C.).

### Data Extraction, Level of Evidence, and Quality Assessment

Data extraction was performed by 4 reviewers (J.W.L., W.W., A.R., and A.M.) using a standardized Excel version 16.78.3 spreadsheet file (Microsoft). Duplicate studies and data were automatically removed by Covidence software.^11^ Study characteristics and patient demographics, management principles (eTable 3 in Supplement 1), and adjusted variables for outcome assessments (eTable 4 in Supplement 1), and all outcomes of interest were extracted using an Excel file. Both unadjusted odds ratios (ORs) and adjusted ORs (aORs) with 95% CIs, if provided, were collected for outcomes assessing association. In the event an unadjusted OR was not provided, these values were calculated using the study’s exposure (patients with or without hypotension) and adverse outcome (binary). For consistent data extraction, studies that provided age in median and IQR values were converted to mean and SD using an online calculator.^12^ In studies that divided their total sample and reported outcomes by subgroups, the study was divided into multiple separate entries as substudies in the Excel file to distinguish the outcomes of specific subgroups.

The Newcastle-Ottawa Scale (NOS) was used in quality assessment of cohort studies (eTable 5 in Supplement 1). The quality of studies was graded based on total points: good quality with low risk of bias (≥7 points), moderate quality (5-6 points), and poor quality (≤4 points).^13^ Studies were also designated based on level of evidence.^14^

### Statistical Analysis

Meta-analysis was performed using Stata software version 18.0 (StataCorp). Primary outcomes were calculated by pooling study-specific ORs with 95% CIs using random-effects models. Adjusted effect estimates reported by individual studies were used, which includes adjustment for at least baseline covariates, including age, sex, and severity (eTable 4 in Supplement 1). Incidence of hypotension was derived from observational studies using logit transformation. Heterogeneity was assessed by Cochran *Q*-test and Higgins *I*^2^ value, meta regression of primary outcomes for continuous variables, and subgroup analysis for categorical variables (eTable 6 and eTable 7 in Supplement 1). Meta-analysis was done on both datasets; however, heterogeneity, bias, and meta-regression were only performed for the dataset with no missing data. The effect of substudies on heterogeneity was also explored by multilevel meta-analysis. Publication bias and outliers were visualized using a funnel plot and Galbraith plot, respectively. Sensitivity analysis was performed using a leave-one-out analysis, and comparing study sets based on data completeness, substudies, and risk of bias. To assess for small study effects, Egger and Begg tests were performed, with *P* < .05 values set as significant. To adjust for publication bias, trim-and-fill analysis was performed. For dispersion of effect estimates for future studies, prediction intervals for both unadjusted and adjusted estimates were provided. *P* values were 2-sided, and statistical significance was set at *P* ≤ .05. Data were analyzed from April 4 to September 10, 2024.

## Results

**Figure 1.**
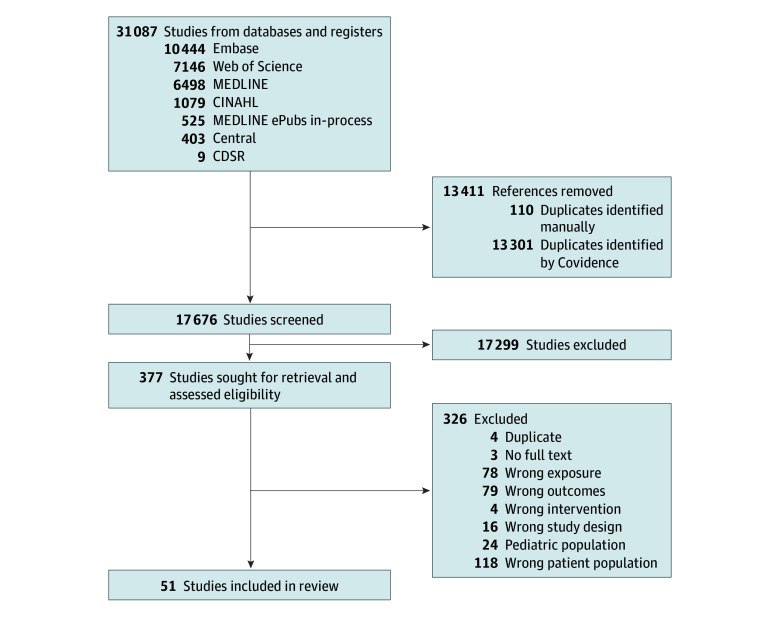
Study Selection Flowchart CDSR indicates Cochrane Database of Systematic Reviews.

A comprehensive search strategy identified a total of 17 676 unique articles. After title and abstract screening, 17 299 articles were deemed irrelevant and excluded. An additional 326 studies were excluded during full-text screening due to wrong patient population (118 studies), wrong exposure (79 studies), wrong outcomes (78 studies), population ages younger than 10 years (24 studies), wrong study design (16 studies), wrong intervention (4 studies), duplicate (4 studies), or no full-text article found (3 studies). In total, 51 unique studies,^2,15,16,17,18,19,20,21,22,23,24,25,26,27,28,29,30,31,32,33,34,35,36,37,38,39,40,41,42,43,44,45,46,47,48,49,50,51,52,53,54,55,56,57,58,59,60,61,62,63^ including 384 329 patients, met criteria for final inclusion (Figure 1). A summarized overview of all included studies is available in the Table. Including substudies, there were 67 studies included for the primary outcome and 52 studies for the secondary outcome. All articles were cohort studies and had a Newcastle-Ottawa Scale score of 5 points or greater. Most studies defined hypotension as SBP less than 90 mm Hg,^2,7,15,16,18,20,21,22,23,24,25,26,28,29,30,31,32,33,34,35,36,37,39,40,45,48,49,50,51,52,53,54,55,57,58,60,61,63^ while other studies reported several hypotension thresholds, ranging from 60 to 120 mm Hg.^17,27,38,43,51,52,56^ Only 1 study^46^ used an individualized hypotension definition (<20% baseline). Most studies reported in-hospital mortality^2,16,17,20,21,22,23,26,29,30,32,38,39,40,41,42,44,47,48,49,50,51,52,53,54,55,56,61^ or 6-month mortality.^19,31,33,34,35,36,45,57,59,60,63^

**Table.** Study Characteristics of All Included Studies

Source	Studies, No.	Country	Design	Sample size, No.	Mean age, y	Male, %	TBI severity	TBI scale	Hypotension threshold, mm Hg	BP measurement setting	Time followed up, d
Aiolfi et al,^15^ 2017	1	US	Cohort	13 188	52	71	Severe	GCS	90	ED	30
Aiolfi et al,^16^ 2018	1	US	Cohort	145 559	55	65	Severe	AIS	90	ED	NR
Asmar et al,^17^ 2021	6	US	Cohort	NR	52, 57	66, 71	Moderate, severe	GCS	70, 90, 110	ED	NR
Brorsson et al,^18^ 2011	1	Sweden	Cohort	38	35	65	Severe	GCS	90	NR	90
Chamoun et al,^19^ 2009	1	US	Cohort	189	37	83	Severe	AIS	NR	ED	180
Chen et al,^20^ 2019	1	US	Cohort	NR	41	71	Severe	GCS	90	ED	NR
Corral et al,^21^ 2012	1	Spain	Cohort	224	38	84	Severe	GCS	90	ICU	NR
Czorlich et al,^22^ 2017	1	Germany	Cohort	442	39	75	Severe	AIS	90	ED, EMS, ICU	NR
DuBose et al,^23^ 2008	1	US	Cohort	16 035	41	70	Severe	AIS	90	ED	NR
Farahvar et al,^24^ 2011	1	US	Cohort	1416	40	78	Severe	GCS	90	ED	14
Farahvar et al,^25^ 2012	1	US	Cohort	1307	40	77	Severe	GCS	90	ED	14
Franschman et al,^26^ 2011	1	The Netherlands	Cohort	274	44	70	Severe	GCS	90	EMS	NR
Fuller et al,^27^ 2014	5	UK	Cohort	5057	45, 47, 48	62, 71	Severe	AIS	70, 80, 90, 100, 110	ED	30
Hartl et al,^28^ 2008	1	US	Cohort	797	NR	NR	Severe	GCS	90	ED	14
Hasanin et al,^29^ 2016	1	Egypt	Cohort	50	31	90	Severe	GCS	90	ED	NR
Heppekcan et al,^30^ 2019	1	Turkey	Cohort	100	32	NR	Severe	GCS	90	ED	NR
Herrera-Melero et al,^31^ 2015	1	Spain	Cohort	629	31	85	Severe	GCS	90	ED	180
Huang et al,^32^ 2022	2	UK	Cohort	182, 234	58, 62	66	Moderate, Severe	GCS	90	ED	NR
Hukkelhoven et al,^33^ 2005	1	The Netherlands	Cohort	2208	33	79	Moderate, severe	GCS	90	EMS	180
Jacobs et al,^34^ 2013	1	The Netherlands	Cohort	700	44	71	Moderate, severe	GCS	90	ED, EMS, ICU	180
Kamal et al,^35^ 2016	1	India	Cohort	1026	31	87	moderate, severe	GCS	90	ED	180
Kamal et al,^36^ 2021	1	India	Cohort	946	31	87	Severe	GCS	90	ED	180
Khalili et al,^37^ 2017	1	Iran	Cohort	307	35	88	Severe	AIS	90	ED	17
Kim et al,^38^ 2018	2	UK	Cohort	99	36	72	Severe	GCS	90, 110	ED	NR
Krishnamoorthy et al,^39^ 2015	1	US	Cohort	41 590	61	64	Severe	AIS	90	ED	NR
Lenstra et al,^40^ 2020	1	The Netherlands	Cohort	198	40	75	Severe	GCS	90	ICU	NR
Manley et al,^2^ 2001	1	US	Cohort	107	46	75	Moderate, severe	GCS	90	ED	NR
Merck et al,^41^ 2019	1	US	Cohort	863	35	74	Moderate, severe	GCS	100	ICU	14
Muehlschlegel et al,^42^ 2013	1	US	Cohort	881	35	70	Moderate, severe	GCS	100	ICU	NR
Newgard et al,^43^ 2015	3	US, Canada	Cohort	1239	37	77	Severe	GCS	90, 105, 120	EMS	28
Para et al,^44^ 2018	1	India	Cohort	70	36	6	Severe	GCS	80	ICU	NR
Petroni et al,^45^ 2010	1	Argentina	Cohort	148	34	81	Severe	GCS	90	ED	180
Pin-On et al,^46^ 2017	4	Thailand	Cohort	100	42	84	Moderate, severe	GCS	<20% baseline, 90	OR (induction), OR (intraoperative)	2
Rauch et al,^47^ 2021	1	Italy	Cohort	142	43	NR	Severe	AIS	110	EMS, ED	NR
Rice et al,^7^ 2023	3	US	Cohort	12 582	40, 41, 45	70	Severe	AIS	90	ED, EMS	14
Schellenberg et al,^48^ 2021	1	US	Cohort	4997	52	NR	Moderate	GCS	90	ED	NR
Shafi et al,^49^ 2005	2	US	Cohort	7601, 1185	35, 37	73, 77	Severe	GCS	90	ED, EMS	NR
Shibahashi et al,^50^ 2017	1	Japan	Cohort	24 833	72	67	Severe	AIS	90	ED	NR
Shibahashi et al,^51^ 2018	6	Japan	Cohort	12 537	59	70	Severe	GCS	70, 80, 90, 100, 110	ED	NR
Shibahashi et al,^52^ 2021	6	Japan	Cohort	30 451	59	69	Severe	AIS	70, 80, 90, 100, 110	EMS	NR
Song et al,^53^ 2023	1	Korea	Cohort	5640	55	72	Severe	AIS	90	EMS	NR
Spaite et al,^54^ 2017	1	US	Cohort	7521	40	29	Severe	AIS	90	ED, EMS	NR
Spaite et al,^55^ 2017	1	US	Cohort	13 151	45	69	Severe	AIS	90	EMS	NR
Spaite et al,^56^ 2017	13	US	Cohort	3844	35	67	Severe	AIS	60, 65, 70, 75, 80, 85, 90, 95, 100, 105, 110, 115, 120	EMS	NR
Spaite et al,^57^ 2022	1	US	Cohort	12 169	44	70	Severe	AIS	90	EMS	180
Tohme et al,^58^ 2014	1	Switzerland	Cohort	589	53	25	Severe	AIS	90	EMS	14
Utomo et al,^59^ 2009	1	Australia	Cohort	428	NR	55	Moderate, severe	AIS	100	ED	180
Vos et al,^60^ 2010	1	the Netherlands	Cohort	79	47	72	Moderate, severe	GCS	90	NR	180
Yeung et al,^61^ 2011	1	China, Australia	Cohort	2979	28	79	Moderate, severe	AIS	90	ICU	NR
Zafar et al,^62^ 2011	1	US	Cohort	7238	54	64	Severe	AIS	120	ED	30
Zeiler et al,^63^ 2021	1	Canada	Cohort	160	47	80	Moderate, severe	GCS	90	NR	180

Abbreviations: AIS, Abbreviated Injury Scale; ED, emergency department; EMS, emergency medical services; GCS, Glasgow Coma Scale; ICU, intensive care unit; NR, not reported or missing data; OR, operating room.

**Figure 2.**
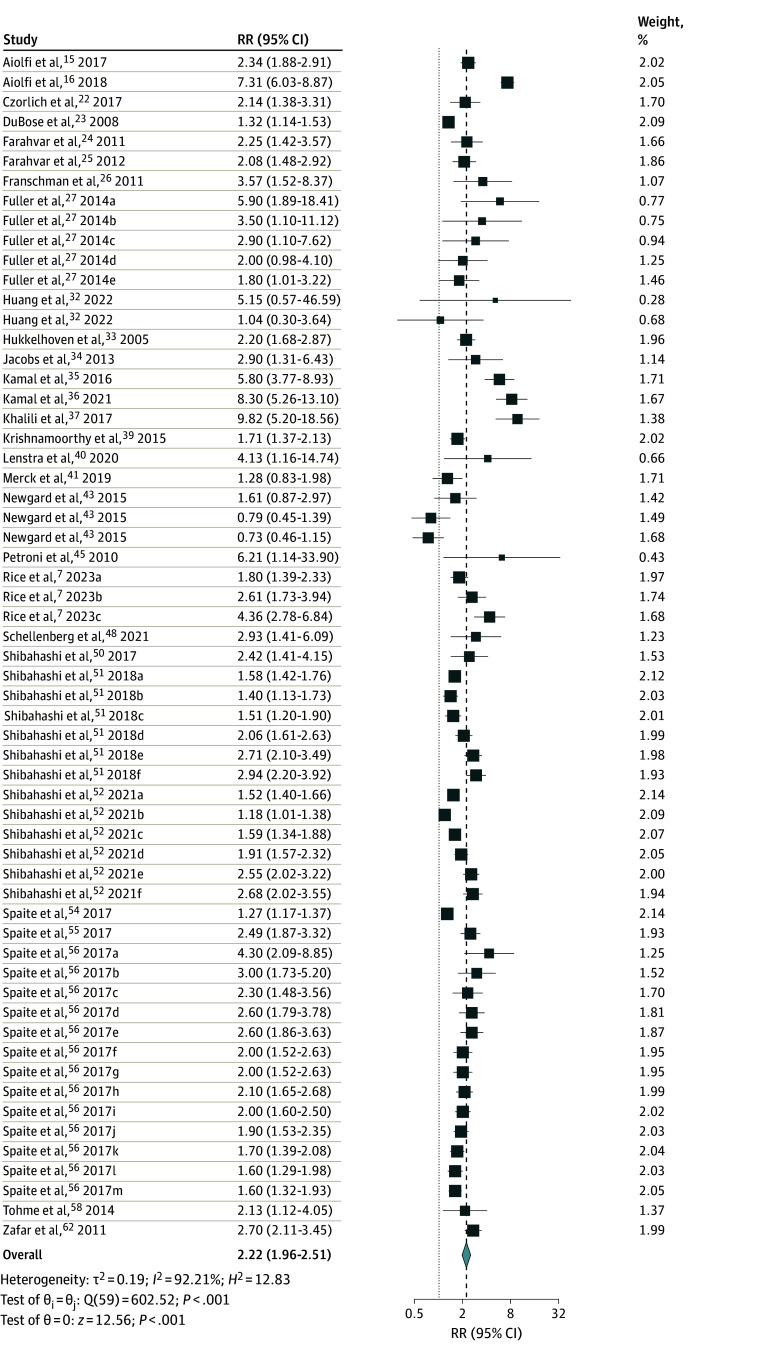
Association of Hypotension With Vegetative State or Mortality in Patients With Moderate to Severe Traumatic Brain Injury Dashed black line indicates the summary measure; dotted gray line, line of no effect; diamond, overall pooled effect estimate of adjusted odds ratio (OR), restricted maximum likelihood random-effect model; sizes of squares, study weight. Substudies from Fuller et al^27^ were divided based on hypotension thresholds of 70 mm Hg (subgroup a), 80 mm Hg (subgroup b), 90 mm Hg (subgroup c), 100 mm Hg (subgroup d), and 110 mm Hg (subgroup e). Substudies from Huang et al^32^ were divided based on moderate traumatic brain injury (subgroup a) and severe traumatic brain injury (subgroup b). Substudies from Newgard et al^43^ were divided based on SBP ranges of less than 90 mm Hg (subgroup a), 90 to 105 mm Hg (subgroup b), and 105 to 120 mm Hg (subgroup c). Substudies from Rice et al^7^ include different blood pressure measurement settings emergency medical services (subgroup a), emergency department (subgroup b), and emergency medical services and emergency department (subgroup c). Substudies from 2 studies by Shibahashi et al^51,53^ were based on thresholds of less than 110 mm Hg (subgroup a), 100 to 109 mm Hg (subgroup b), 90 to 99 mm Hg (subgroup c), 80 to 89 mm Hg (subgroup d), 70 to 79 mm Hg (subgroup e), less than 70 mm Hg (subgroup f). Substudies from Spaite et al^56^ include blood pressure thresholds increments of 5 mm Hg from less than 60 mm Hg (subgroup a) to less than 120 mm Hg (subgroup m).

Mortality was reported as the primary outcome in all studies. The pooled analysis of 51 studies (171 744 patients) revealed a significantly increased odds of mortality (crude OR, 3.82; 95% CI, 3.04-4.81; *P* < .001; *I*^2^ = 96.98%) in patients with hypotension and moderate to severe TBI (eFigure 1 in Supplement 1). Additionally, we calculated odds of mortality from studies reporting adjusted confounders (aOR, 2.22; 95% CI, 1.96-2.51; *P* < .001; *I*^2^ = 92.21%) (60 studies; 350 662 patients) (Figure 2).

**Figure 3.**
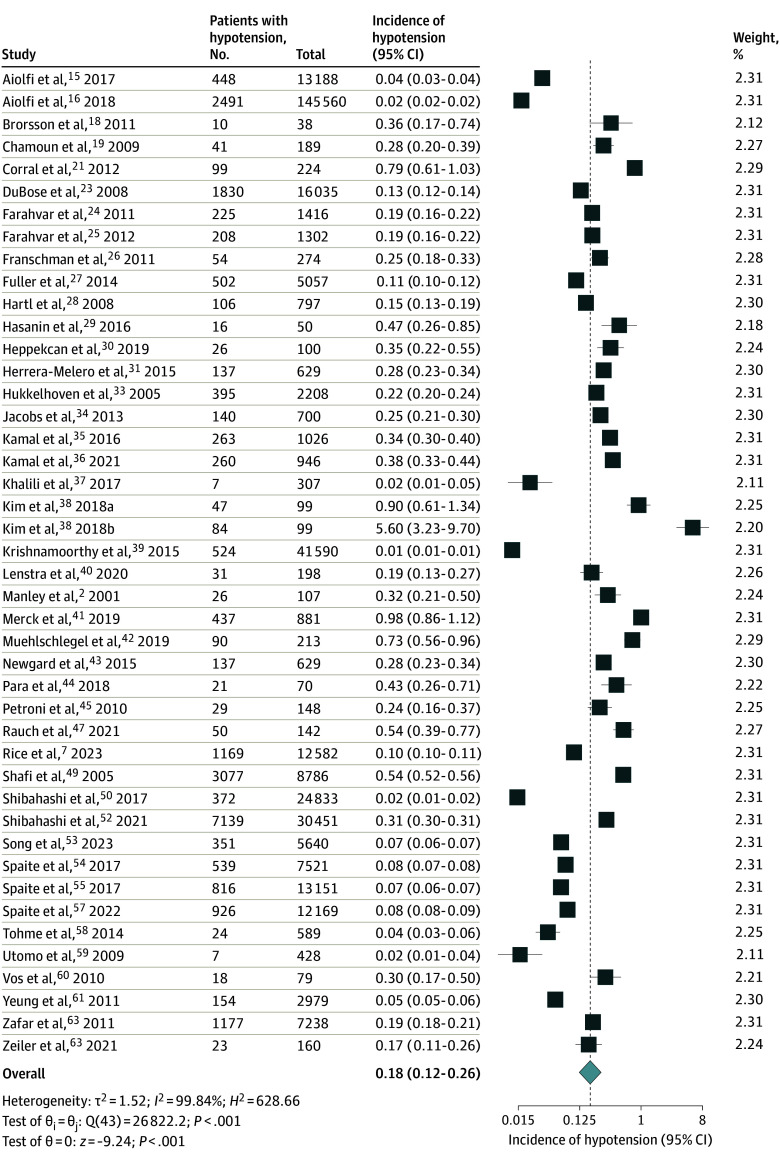
Incidence of Hypotension in Patients With Moderate to Severe Traumatic Brain Injury Dashed line indicates the summary measure; diamond, overall pooled effect estimate of incidence, restricted maximum likelihood random-effect model; squares, individual estimates; sizes of squares, study weight. Substudies from Kim et al^38^ were divided based on blood pressure thresholds at less than 90 mm Hg (subgroup a) and less than 110 mm Hg (subgroup b).

Incidence of hypotension was reported in 44 studies (360 729 patients).^2,7,15,16,18,19,21,23,24,25,26,27,28,29,30,31,33,34,35,36,37,38,39,40,41,42,43,44,45,47,49,50,51,52,53,56,57,58,59,60,61,62,63^ Pooled analysis revealed an 18% incidence (95% CI, 12%-26%) (*P* = .001; *I*^2^ = 99.84%) (Figure 3). The incidence was consistently high across various subgroups and BP cutoffs (eFigure 2 in Supplement 1). There was variability in defining a hypotension cutoff among studies. Specifically, the incidence was 15% (95% CI, 11%-22%) when hypotension was defined as SBP lower than 90 mm Hg (*P* < .001) (35 studies). Patients with multiple-trauma TBI also had a higher incidence of hypotension (21%) compared with patients with isolated TBI (11%).

**Figure 4.**
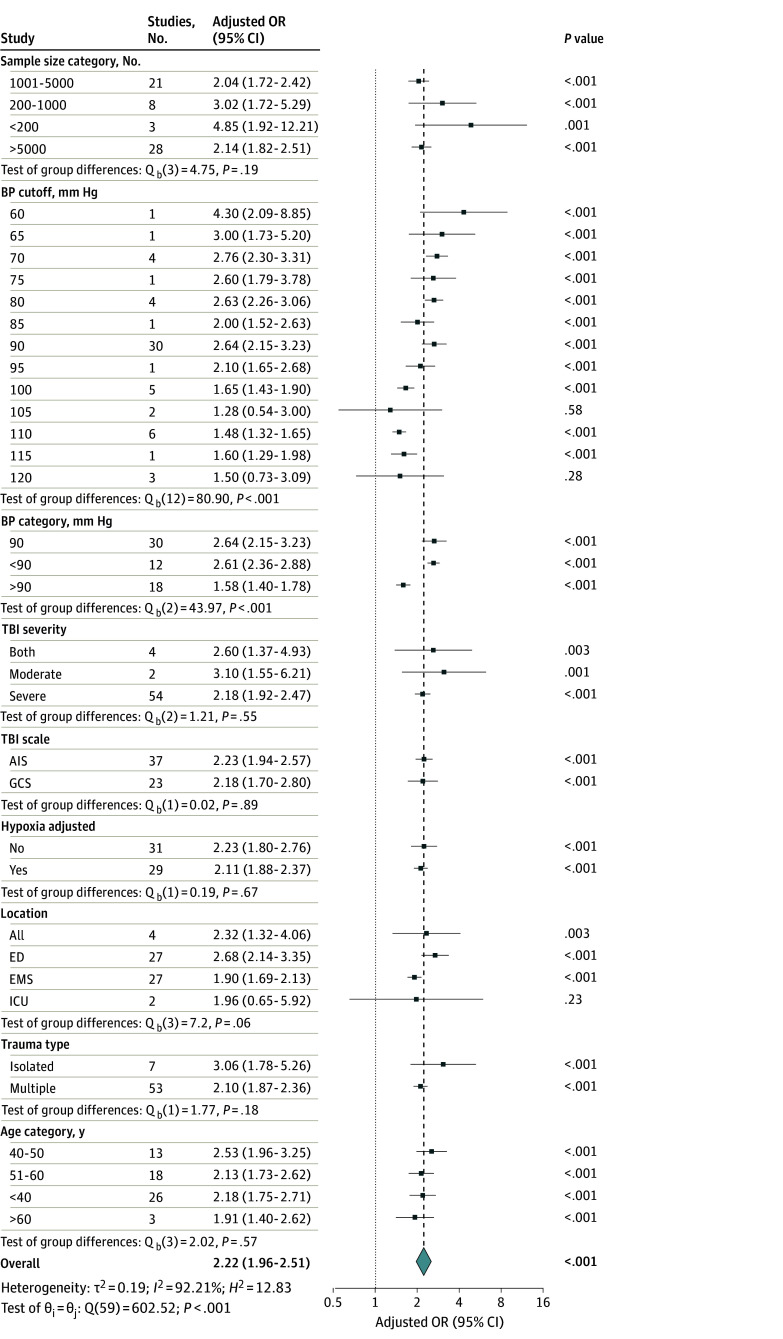
Association of Hypotension With Vegetative State or Mortality in Patients With Moderate to Severe Traumatic Brain Injury Based on Subgroups Dashed black line indicates the summary measure; dotted line, line of no effect; diamond, overall pooled effect estimate of adjusted odds ratio (OR), restricted maximum likelihood random-effect model. AIS indicates Abbreviated Injury Scale; BP, blood pressure; ED, emergency department; EMS, emergency medical services; GCS, Glasgow Coma Scale; ICU, intensive care unit.

Subgroup meta-analysis revealed that the adjusted (Figure 4) and crude (eFigure 3 in Supplement 1) risk of mortality varied across subgroups but indicated a consistent positive association between mortality and hypotension in patients with TBI. Specifically, an SBP less than 90 mm Hg was associated with a 2.64-fold increased risk of mortality (aOR, 2.64; 95% CI, 2.15-3.23; *P* < .001). In comparison, SBP greater than 90 mm Hg was associated with lower odds of mortality (aOR, 1.58; 95% CI, 1.40-1.78; *P* < .001). Adjustment for hypoxia as a comorbidity decreased the aOR from 2.23 (95% CI, 1.80-2.76) to 2.11 (95% CI, 1.88-2.37). Most studies used SBP measurements from the emergency department (ED) or emergency medical services (EMS). Measurement in the ED was associated with increased mortality (aOR, 2.68; 95% CI, 2.14-3.35; *P* < .001) compared with EMS (aOR, 1.90; 95% CI, 1.69-2.13; *P* < .001). Patients with isolated TBI had an increased risk of mortality (aOR, 3.06; 95% CI, 1.78-5.26; *P* < .001) compared with patients with multiple-trauma TBI (aOR, 2.10; 95% CI, 1.87-2.36; *P* < .001). Tests of group differences across 6 categories revealed statistically significant differences between groups based on BP threshold categories and location of BP measurement (Figure 4).

Subgroup analysis and meta-regression analysis elucidated significant heterogeneity imposed by various categorical and continuous variables (eTable 6 and eTable 7 in Supplement 1). A leave-one-out sensitivity meta-analysis did not indicate that the exclusion of any single study skewed the crude OR or aOR (eTable 8 and eTable 9 in Supplement 1). Heterogeneity and effect size based on various models are summarized in eTable 10 in Supplement 1. Inclusion of 7 studies with incomplete sample size data had similar results (aOR, 2.17; 95% CI, 1.93-2.44). The 95% prediction interval indicated that the dispersion of effect estimates for future studies was between 0.78 and 17.93 for crude OR and 0.93 and 5.31 for aOR.

There was some evidence of publication bias against studies with small sample sizes reporting aORs (eFigures 4 and eFigure 5 in Supplement 1). Trim and fill analysis suggested that imputation of 14 studies with smaller sample sizes results in a decreased aOR for mortality in patients with TBI and hypotension (aOR, 1.87; 95% CI, 1.61-2.16) (eFigure 6 in Supplement 1).

There was limited reporting of duration of hypotension, hospital stay, and intensive care unit stay. Only 53% of studies reported data on hypotension management, of which 96% of studies used fluids, 56% of studies used vasopressors, and 19% of studies involved transfusions (eTable 7 in Supplement 1).

## Discussion

With a total sample size of more than 380 000 patients across 51 unique studies, our systematic review and meta-analysis provides empirical evidence of the adverse outcomes associated with hypotension after moderate to severe TBI injury. The crude OR suggests an almost 4-fold risk of mortality, and the aOR found more than 2-fold risk. While hypotension is a well-known risk factor for poorer outcomes in trauma settings (including head injuries),^64^ to our knowledge, this is the first review that provides comprehensive and aggregate empirical data of mortality and incidence of hypotension.

Using subgroup analysis of the primary outcome, we identified important underlying variables contributing to mortality of patients with TBI and hypotension, supporting the multifactorial nature of hypotension in patient outcomes. Hypotension using a 90 mm Hg threshold showed significantly increased 2.64-fold odds for mortality. Meanwhile, a BP threshold set higher than 90 mm Hg showed mortality odds of 1.58, a 60% reduction, once again emphasizing the need to avoid lower than 90 mm Hg for treatment of patients with TBI. To specifically assess the appropriateness of Brain Trauma Foundation guidelines for BP management, an analysis of SBP threshold of 100 mm Hg and 110 mm Hg was performed and demonstrated ORs of 1.65 and 1.48, respectively, representing a significant improvement in odds compared with a threshold of 90 mm Hg. Thus, current guidelines seem to be appropriate and recommendable based on the analysis. Although there were 2 separate classification systems (Abbreviated Injury Scale [AIS] and Glasgow Coma Scale [GCS]) assessing TBI severity, the odds for mortality showed no significant differences. Interestingly, isolated TBI groups showed higher mortality compared with multiple-trauma TBI. In a study comparing mortality rates between patients with multiple-trauma TBI and isolated TBI, Niemmeyer et al^65^ did not find a significant difference in mortality rate between isolated and multiple-trauma TBI (35% vs 24%; *P* = .06). Due to the absence of other injuries, isolated TBI–related hypotension is more likely to be attributed by neurogenic mechanisms. Neurogenic hypotension may be associated with poorer outcomes due to direct insult to brain structures, such as the brainstem or hypothalamus, which can lead to subsequent systemic changes, including loss of sympathetic tone, and catecholamine surges potentially causing arrythmias and pulmonary edemas.^66,67^ The setting of BP measurement (ED vs EMS) also showed differences in mortality. Hypotension measured in the ED yielded a much higher OR compared with that measured by EMS (aOR, 2.68 vs 1.90). Hypotension measured in the ED might include patients with a sustained or prolonged hypotension compared with a more transient hypotension measured in patients being treated by EMS, which may resolve before reaching the hospital. Indeed, Kumar et al^68^ showed a significantly increased risk of mortality in patients with septic shock with a longer duration of hypotension, which may explain our differences in mortality outcomes. Overall, our subgroup analyses raise important considerations regarding clinical factors that contribute to mortality in patients with TBI and hypotension. Studies exhibit a wide range of mortality risk, such as Newgard et al^43^ (OR, 0.73) and Khalil et al^37^ (OR, 9.82). The lower odds can be explained by the higher BP threshold used in the study by Newgard et al^43^ (120 mm Hg). Alternatively, the higher association with mortality can be justified by understanding the inclusion criteria used by Khalil et al^37^: patients with isolated TBI only, severe TBI, and BP measurement in the ED—all factors that were associated with higher mortality.

Pooled analysis revealed a striking overall hypotension incidence of 18%. However, there was variability among the studies in defining hypotension within the context of TBI. Specifically, the incidence of hypotension was lower (15%) when hypotension was defined as SBP lower than 90 mm Hg. Interestingly, the incidence of hypotension was much lower when classified according to AIS vs GCS (7% vs 35%). Interestingly, while isolated TBI was associated with greater risk of mortality, the incidence of hypotension was lower compared with multiple-trauma TBI, likely due to the higher prevalence of hemorrhagic hypotension. Incidence of hypotension showed differences in various subgroups (age <40 years, GCS score, threshold level, multiple trauma). This knowledge should be used to guide the urgency and aggressiveness of BP control as part of TBI management.

### Limitations

Our study has some limitations. First, none of the included studies reported data on vegetative state. Another limitation was the lack of uniformity of reported data among included studies. Some studies may not have adjusted for known factors associated with TBI-related mortality, such as pupillary score, motor score, computed tomography classification, and glucose, resulting in reporting ORs with potential confounders. Similarly, another limitation was the limited data on hypotension duration and management strategies, as both of these factors can affect mortality outcomes. There was also high heterogeneity in our mortality outcome data, reducing the generalizability and applicability of our findings. Most studies in our meta-analysis did not exclude patients with preexisting hypotension. Patients with preexisting hypotension may exhibit distinct outcomes from those who develop hypotension secondary to TBI. Thus, this subset of patients included within our analysis may serve as potential confounders.

We aimed to address these limitations using a methodical approach to evaluate heterogeneity via subgroup analysis and meta-regression. We found that the high degree of heterogeneity (>75%) was most likely attributed to the differences in study design, baseline characteristics, sample size, and correlation of exposure (substudies) and effect size. Hypoxia is a known secondary injury in TBI that contributes to worse outcomes.^2^ Including adjusted effect estimates for hypoxia, other potential confounders, and publication bias still resulted in 1.87 times higher odds of mortality in these patients with hypotension. By combining the outcomes of 51 studies with a total sample size of more than 380 000 patients, our study reinforces the observed association between hypotension in patients with TBI and mortality.

Future research should report more comprehensive data stratified by age, duration of hypotension, and number of hypotensive insults. This would allow for more in-depth analysis of patient outcomes stratified by the patient characteristics and hypotension event, which would be valuable to guide clinical decision-making. Management strategies should also be reported for all studies as this can majorly affect patient outcomes. Type and dosage of fluid can provide different outcomes when resuscitating patients with brain injury.^69^ However, information regarding type, dosage, and timing of fluid were not reported in most studies and should be provided in future studies.

## Conclusions

In this systematic review and meta-analysis of more than 380 000 patients with moderate to severe TBI, we found significant association of mortality following hypotension. Our findings suggest that management guidelines should reinforce the need to aggressively treat hypotension and maintain BP within reference range in patients with TBI to prevent mortality.

## Notes

Supplement 1. **eAppendix.** Definitions of TBI and Hypotension **eTable 1.** Search Strategy **eTable 2.** Definition of Continuous and Categorical Variables **eTable 3.** Reported Management Strategies for Hypotension **eTable 4.** Adjusted Factors for Calculation of Adjusted Odds Ratios **eTable 5.** Quality Assessment Using Newcastle-Ottawa Scale for Cohort Studies **eTable 6.** Heterogeneity Analysis for Continuous Variables **eTable 7.** Heterogeneity Analysis for Categorical Variables **eTable 8.** Leave-One-Out Analysis for Studies Reporting Adjusted OR **eTable 9.** Leave-One-Out Analysis for Studies Reporting Crude OR **eTable 10.** Heterogeneity Analysis for Substudies Using Multilevel Meta-Analysis **eFigure 1.** Forest Plot Showing Crude Association of Hypotension on Adverse Outcome in Patients With Moderate to Severe Traumatic Brain Injury **eFigure 2.** Forest Plot Showing Incidence of Hypotension by Subgroups in Patients With Moderate to Severe Traumatic Brain Injury **eFigure 3.** Forest Plot Showing Association of Hypotension on Adverse Outcome in Patients With Moderate to Severe Traumatic Brain Injury **eFigure 4.** Funnel Plot for Publication Bias **eFigure 5.** Galbraith Plot for Exhibiting Outliers **eFigure 6.** Trim Fill Funnel Plot for Publication Bias

Supplement 2.
Data Sharing Statement
