# Supplementary material for: Hypotension and Adverse Outcomes in Moderate to Severe Traumatic Brain Injury: A Systematic Review and Meta-Analysis
Source: JAMA Netw Open. 2024 Nov 11;7(11):e2444465. doi: 10.1001/jamanetworkopen.2024.44465 (PMC11555550; doi:10.1001/jamanetworkopen.2024.44465)
Supplement: Supplement 1. — eAppendix. Definitions of TBI and Hypotension eTable 1. Search Strategy eTable 2. Definition of Continuous and Categorical Variables eTable 3. Reported Management Strategies for Hypotension eTable 4. Adjusted Factors for Calculation of Adjusted Odds Ratios eTable 5. Quality Assessment Using Newcastle-Ottawa Scale for Cohort Studies eTable 6. Heterogeneity Analysis for Continuous Variables eTable 7. Heterogeneity Analysis for Categorical Variables eTable 8. Leave-One-Out Analysis for Studies Reporting Adjusted OR eTable 9. Leave-One-Out Analysis for Studies Reporting Crude OR eTable 10. Heterogeneity Analysis for Substudies Using Multilevel Meta-Analysis eFigure 1. Forest Plot Showing Crude Association of Hypotension on Adverse Outcome in Patients With Moderate to Severe Traumatic Brain Injury eFigure 2. Forest Plot Showing Incidence of Hypotension by Subgroups in Patients With Moderate to Severe Traumatic Brain Injury eFigure 3. Forest Plot Showing Association of Hypotension on Adverse Outcome in Patients With Moderate to Severe Traumatic Brain Injury eFigure 4. Funnel Plot for Publication Bias eFigure 5. Galbraith Plot for Exhibiting Outliers eFigure 6. Trim Fill Funnel Plot for Publication Bias [file jamanetwopen-e2444465-s001.pdf]

Supplemental Online Content

Lee JW, Wang W, Rezk A, et al. Hypotension and adverse outcomes in moderate to severe traumatic brain injury: a systematic review and meta-analysis. JAMA Netw Open. 2024;7(11):e2444465. doi:10.1001/jamanetworkopen.2024.44465

eAppendix. Definitions of TBI and Hypotension
eTable 1. Search Strategy
eTable 2. Definition of Continuous and Categorical Variables
eTable 3. Reported Management Strategies for Hypotension
eTable 4. Adjusted Factors for Calculation of Adjusted Odds Ratios
eTable 5. Quality Assessment Using Newcastle-Ottawa Scale for Cohort Studies
eTable 6. Heterogeneity Analysis for Continuous Variables
eTable 7. Heterogeneity Analysis for Categorical Variables
eTable 8. Leave-One-Out Analysis for Studies Reporting Adjusted OR
eTable 9. Leave-One-Out Analysis for Studies Reporting Crude OR
eTable 10. Heterogeneity Analysis for Substudies Using Multilevel Meta-Analysis
eFigure 1. Forest Plot Showing Crude Association of Hypotension on Adverse Outcome in Patients With Moderate to Severe Traumatic Brain Injury
eFigure 2. Forest Plot Showing Incidence of Hypotension by Subgroups in Patients With Moderate to Severe Traumatic Brain Injury
eFigure 3. Forest Plot Showing Association of Hypotension on Adverse Outcome in Patients With Moderate to Severe Traumatic Brain Injury
eFigure 4. Funnel Plot for Publication Bias
eFigure 5. Galbraith Plot for Exhibiting Outliers
eFigure 6. Trim Fill Funnel Plot for Publication Bias

This supplemental material has been provided by the authors to give readers additional information about their work.

© 2024 Lee JW et al. *JAMA Network Open.*

eAppendix: Definitions of TBI and hypotension.

TBI is defined as any neurological insult secondary to penetrating or blunt trauma to the head. TBI severity is categorized into mild, moderate, or severe. TBI severity is commonly assessed using one of two scoring systems (TBI Scale); the abbreviated injury (AIS) scores and/or Glasgow coma scale (GCS) scores. AIS score of 2,3+, or a GCS score within 9-12, and 3-8 corresponds to moderate and severe TBI respectively.[67,68]

Hypotension in the setting of TBI can be defined using different measurements. SBP is a common mode of measurement. For the purpose of this study, hypotension was defined as a SBP < 90mmHg. Other hypotension thresholds were included if study authors utilized a different threshold for hypotension (i.e., <110 mmHg, <100 mmHg, etc.) and/or in cases where study authors identified a SBP threshold which had a significant association with adverse outcome(s). Setting of hypotension measurement is defined as the location of measurement within patient management and care of TBI which include: emergency medical services (EMS), hospital (ED) arrival, and during ICU care.

© 2024 Lee JW et al. *JAMA Network Open.*

eTable 1: Search Strategy

MEDLINE
Ovid MEDLINE(R) 1946 to April 04, 2024

#	Searches
1	exp Hypotension/
2	exp Cerebrovascular Circulation/
3	Blood Pressure Determination/
4	exp Blood Pressure/
5	(arter* pressure? adj8 (diastolic or systolic)).mp.
6	(blood pressure? and (low* or abnormal* or challeng* or deficien* or drop*4 or restor*)).mp.
7	brain blood flow*.mp.
8	cerebral perfusion pressure?.mp.
9	cerebral* blood flow*.mp.
10	cerebral* circulation?.mp.
11	cerebrovascular* circulation*.mp.
12	diastolic pressure?.mp.
13	hypotensi*.mp.
14	pulse pressure?.mp.
15	systolic pressure?.mp.
16	"60 millimeter? of mercury".mp.
17	"60 mm Hg?".mp.
18	"60 mmHg?".mp.
19	"60mmHg?".mp.
20	"90 millimeter? of mercury".mp.
21	"90 mm Hg?".mp.
22	"90 mmHg?".mp.
23	"90mmHg?".mp.
24	("90/60" and (diastolic or systolic)).mp.
25	or/1-24 [Hypotension & related Terms]
26	exp Brain Injuries/

© 2024 Lee JW et al. *JAMA Network Open.*

27	exp Brain Concussion/
28	Coma, Post-Head Injury/
29	Contrecoup Injury/
30	Craniocerebral Trauma/
31	Glasgow Coma Scale/ and (exp Accidents/ or (accident* or injur* or insult? or event? or trauma*).mp.)
32	Glasgow Outcome Scale/ and (exp Accidents/ or (accident* or injur* or insult? or event? or trauma*).mp.)
33	exp Head Injuries, Closed/
34	Head Injuries, Penetrating/
35	exp Intracranial Hemorrhage, Traumatic/
36	Persistent Vegetative State/
37	exp Skull Fractures/
38	Subarachnoid Hemorrhage, Traumatic/
39	((brain? adj3 injur*) and (accident* or trauma*)).ti,ab.
40	(closed adj3 head adj2 injur*).mp.
41	(closed adj3 head adj2 traum*).mp.
42	(coup adj3 injur*).mp.
43	(craniocerebral* adj1 trauma*).mp.
44	(cranio-cerebral* adj1 trauma*).mp.
45	(diffuse adj1 axon* adj1 injur*).mp.
46	(Glasgow adj2 coma* adj10 (accident* or injur* or insult? or event? or trauma*)).mp.
47	(head adj2 injur*).mp.
48	(head adj2 trauma*).mp.
49	(head? adj1 impact*).mp.
50	(injur* adj3 (head? or brain* or cerebr* or cerebell* or cranial* or cranium or skull?)).mp.
51	(insult* adj3 (head? or brain* or cerebr* or cerebell* or cranial* or cranium or skull?)).mp.
52	(second* adj2 impact* adj1 syndrom*).mp.
53	(skull* adj2 fractur*).mp.
54	(subarachnoid* adj2 h?emorrhage? adj10 (accident* or injur* or insult? or crash* or traffic* or trauma*)).mp.
55	(trauma* adj4 (head? or brain* or cerebr* or cerebell* or cranial* or cranium or skull?)).mp.

© 2024 Lee JW et al. *JAMA Network Open.*

56	(traumatic* adj2 encephalopath*).mp.
57	commotio cerebri.mp.
58	concuss*.mp.
59	contre-coup.mp.
60	(contre-coup* and (head? or brain* or cranial* or cranium or skull?)).mp.
61	(contrecoup* and (head? or brain* or cranial* or cranium or skull?)).mp.
62	(contusion* and (head? or brain* or cerebr* or cerebell* or cranial* or cranium or skull?)).mp.
63	coup-contrecoup injur*.mp.
64	((mtbi or mtbis) and (head? or brain* or cerebr* or cerebell* or cranial* or cranium or skull?)).mp.
65	(neuro-trauma* and (head? or brain* or cerebr* or cerebell* or cranial* or cranium or skull?)).mp.

© 2024 Lee JW et al. *JAMA Network Open.*

eTable 2: Definition of continuous and categorical variables.

Variable	Definition
**Categorical**	
Sample size group	• Studies were divided into four groups based on sample size for subgroup and sensitivity analysis (<200, 201-1000, 1001-5000, >5000)
TBI severity	• TBI injury was categorized by severity based on GCS and AIS score thresholds as outlined in the definitions section. TBI severity was categorized as moderate only, severe only, and moderate-severe
TBI scale	• Degree and severity of TBI injury is often classified using GCS or AIS scoring systems. TBI scale was categorized as either GCS or AIS depending on the scale used by the study to define moderate, severe, or moderate-severe TBI
Location	• The location/setting initial blood pressure measurement was performed in the study. Blood pressure setting was categorized into emergency medical services/ambulance/pre-hospital (EMS), emergency department (ED), intensive care unit (ICU) or intraoperative (IOP). In the event study measured blood pressure in EMS, ED and ICU settings, setting was categorized as ALL
Trauma type	• Studies were categorized based on the type of trauma patients included in the study. Type was categorized as isolated if study only included traumatic brain injury patients with no other injury site on the body. Alternatively, studies were categorized as polytrauma if it included TBI patients with other body injury sites
Blood pressure category	• Thresholds were categorized for subgroup analysis (<90 mmHg, 90 mmHg, >90 mmHg)
Age category	• Studies were divided into four groups based on mean age (<40, 40-50, 50-60, >60)
Hypoxia adjusted	• Studies in which hypoxia was measured and adjusted for calculating their adjusted odds ratios
**Continuous**	
Total sample size	• Total sample size of the study
Mean age	• Mean age of the study
Male %	• Proportion of males which make up the total sample size
Blood pressure cutoff	• The range of blood pressure between 0 and the study's stated threshold

© 2024 Lee JW et al. *JAMA Network Open.*

eTable 3: Reported management strategies for hypotension

Study	Hypotension management
Aiolfi 2017	N/A
Aiolfi 2018	N/A
Asmar 2021	N/A
Brorsson 2011	N/A
Chamoun 2009	1, 2
Chen 2019	N/A
Corral 2012	1, 2
Czorlich 2017	1
DuBose 2008	N/A
Farahvar 2011	1
Farahvar 2012	1
Franschman 2011	1, 2
Fuller 2014	N/A
Hartl 2008	1, 2
Hasanin 2016	N/A
Heppekcan 2019	1, 2
Herrera-Melero 2015	1, 2
Huang 2022	N/A
Hukkelhoven 2005	N/A
Jacobs 2013	N/A
Kamal 2016	N/A
Kamal 2021	N/A
Khalili 2017	1, 2
Kim 2018	1, 2
Krishnamoorthy 2015	N/A
Lenstra 2020	N/A
Manley 2001	1, 2, 3
Merck 2019	1, 2, 3
Muehlschlegel 2013	1, 2
Newgard 2015	1, 3
Para 2018	1, 2
Petroni 2010	1, 2
Pin-On 2017	1, 3
Rauch 2021	1, 2
Rice 2023	1
Schellenberg 2021	N/A
Shafi 2005	1
Shibahashi 2017	1
Shibahashi 2018	1
Shibahashi 2021	1
Song 2023	3
Spaite 2017[a]	N/A
Spaite 2017[b]	N/A
Spaite 2017[c]	N/A
Spaite 2022	N/A
Tohme 2014	1
Utomo 2009	N/A
Vos 2010	N/A
Yeung 2021	N/A
Zafar 2011	N/A
Zeiler 2021	1, 2

Legend: 1 = fluids, 2 = vasopressor, 3 = transfusion. [a,b,c] indicates separate studies from same author and year,

© 2024 Lee JW et al. *JAMA Network Open.*

eTable 4: Adjusted factors for calculation of adjusted odds ratios

Study	Adjusted factors
Aiolfi 2017	• Age, sex, comorbidities, hypotension, tachycardia, GCS, AIS3, AIS4, AIS5, prehospital intubation, level 1 trauma center, transport time, HEMS
Aiolfi 2018	• Age, hypotension on admission, GCS, prehospital intubation, H-AIS 4, H-AIS 5
Asmar 2021	• Patient demographics, vital signs, injury parameters
Chen 2019	• Age, sex, race, insurance status, transport mode, mechanism of injury, ISS, prehospital SBP, admission SBP, need for urgent operation, mechanical ventilation, in-hospital complications, and trauma center level
Czorlich 2017	• Age, BMI, GCS, injury pattern, pre-hospital CPR, blood pressure, ISS, head injury
DuBose 2008	• Age, ACS level, mechanism, ISS, hypotension, and GCS
Farahvar 2011	• Age, day 1 hypotension status, day 1 pupillary status, initial gcs score, CT scan results
Farahvar 2012	• Age, hours of ICP > 25 mm Hg in the first 24 hours, GCS score, pupillary abnormalities, CT findings (partial open/closed cisterns or ≥0·5-cm midline shift)
Franschman 2011	• Age, pupillary reactivity, and GCS, hypoxia, hypotension, ISS
Fuller 2014[a]	• Age, ISS, admission GCS, Marshall Score, hypoxia, traumatic subarachnoid hemorrhage
Huang 2022	• Patient demographics, injury parameters, vital signs
Hukkelhoven 2005	• Age, motor score, pupillary reactivity, hypoxia, hypotension, CT parameters
Jacobs 2013	• Demographic, clinical and CT characteristics
Kamal 2016	• Demographics (age, gender), clinical severity (the motor GCS at admission, pupillary reactivity, limb movement and major extracranial injuries), secondary insult (hypotension) and various CT findings (midline shift, subdural haematoma, epidural haematoma (EDH), basal cistern effaced, presence of traumatic subarachnoid haemorrhage/intraventricular haematoma
Kamal 2021	• Demographics (age, gender), clinical severity (the motor GCS at admission, pupillary reactivity, limb movement and major extracranial injuries), secondary insult (hypotension) and various CT findings (midline shift, subdural haematoma, epidural haematoma (EDH), basal cistern effaced, presence of traumatic subarachnoid haemorrhage/intraventricular haematoma
Khalili 2017	• Age ≥55, selenase, gender, hypotension, GCS ≤8, Rotterdam, IVH, SAH, EDH, SDH, ICH, contusion, neurosurgical intervention, ICU LOS, hospital LOS
Krishnamoorthy 2015	• Age, trauma designation, teaching status, hospital size, gender, admission hypotension, admission GCS, admission ISS, and ventilator requirement during hospitalization, race, insurance status, hospital profit status, number of neurosurgeons at facility, and facility region
Lenstra 2020	• Age, hours of ICP higher than 25 mm Hg in the first 24 hours, GCS score, pupillary abnormalities, CT findings (partial open/closed cisterns or ≥0·5-cm midline shift)
Merck 2019	• O2 Sat, Temperature, ICP, Glucose, INR, Hb, CPP, MAP, Brain tissue oxygen tension, SBP
Newgard 2015	• Age, sex, ISS, head AIS score, initial SBP category, pulse rate category, initial GCS, advanced airway attempts, air transport, site
Petroni 2010	• Age, GCS, pupillary response, hypotension, presence of compression or midline shift on CT findings
Rice 2023	• Age, sex, race, ethnicity, payment source, trauma type (blunt or penetrating), head region, severity score (ICD-9) matched to Abbreviated Injury Scale), ISS, multisystem TBI (anybody region other than head with a severity score of at least 3), intervention of guideline implementation, prehospital hypoxia, prehospital CPR, and treating trauma center prehospital CPR, treating trauma center
Schellenberg 2021	• Patient demographic data (age, gender, and comorbidities), clinical data [prehospital and first ED systolic blood pressure (SBP, mmHg), heart rate (HR, beats per minute (bpm)), and GCS]; injury data [mechanism of injury, level of trauma center, Injury Severity Score (ISS), AIS by body region, and presence of drug or alcohol intoxication]; clinically relevant times (time to first CTH, time to intervention, and time spent in ED)
Shibahashi 2017	• Age; sex; year of admittance; GCS score on arrival at the hospital; comorbidities; hypotension (<90 mm Hg systolic) on arrival; RTS; ISS; whether head CT was performed for initial surveying; nature of the head injury
Shibahashi 2018	• Age, sex, year of hospital admittance, Glasgow Coma Scale score on arrival, Injury Severity Score, major extracranial injuries, maximum head AIS score
Shibahashi 2021	• Age, sex, year of hospital admission, time of day, nature of the injury, prehospital Japan Coma Scale, maximum head AIS score, ISS
Spaite 2017[a]	• Age, sex, race, ethnicity, Injury Severity Score (ISS), and head region injury score (H-AIS), Trauma type (blunt versus penetrating), payment source, treating TC
Spaite 2017[b]	• Age, sex, race, ethnicity, Injury Severity Score (ISS), and head region injury score (H-AIS), interfacility transfer, trauma type (blunt versus penetrating), payment source, treating TC
Spaite 2017[c]	• Age, sex, race, ethnicity, payment source, trauma type (blunt/penetrating), prehospital hypoxia, prehospital intubation, treating trauma center

© 2024 Lee JW et al. *JAMA Network Open.*

eTable 5: Quality assessment using Newcastle-Ottawa Scale for Cohort Studies

Study ID	Author	Year	1	2	3	4	5	6	7	8	9	Quality
#57	Rice et al	2023	1	1	1	1	2	1	0	1	8	Good
#132	Huang et al.	2022	0	1	1	1	1	1	0	1	6	Fair
#184	Yang et al	2022	1	1	1	1	2	1	1	1	9	Good
#325	Rauch et al	2021	1	1	1	1	1	1	0	0	6	Fair
#373	Shibahashi et al	2021	1	1	1	1	1	1	0	1	7	Good
#387	Kamal et al	2021	1	1	1	1	1	1	1	1	8	Good
#416	Gaitanidis et al	2021	1	1	1	1	1	1	1	1	8	Good
#440	Asmar et al	2021	0	1	1	1	1	1	0	1	6	Fair
#444	Zeiler et al	2021	1	1	1	1	1	1	1	1	8	Good
#454	Schellenberg et al	2021	0	1	1	1	0	1	0	1	5	Fair
#713	Heppekcan et al	2019	1	1	1	0	0	1	0	1	5	Fair
#268	Reza Bagheri et al	2021	0	1	1	1	0	1	1	1	6	Fair
#741	Chen et al	2019	0	1	0	1	1	1	0	1	5	Fair
#179	Spaite et al.	2022	1	1	1	1	2	1	0	1	8	Good
#874	Shibahashi et al	2018	1	1	1	1	1	1	0	1	7	Good
#537	Gao et al.	2020	1	1	1	1	1	1	0	1	7	Good
#779	Merck et al.	2019	0	1	1	1	2	1	0	1	7	Good
#787	Pakkanen et al	2019	0	1	1	1	2	1	1	1	8	Good
#999	Aiolfi et al	2018	1	1	1	1	1	1	1	1	8	Good
#1025	Kim et al	2018	1	0	1	1	0	1	1	1	6	Fair
#1093	Czorlich et al	2017	1	0	1	1	0	1	1	1	6	Fair
#1102	Shibahashi et al	2017	1	0	1	1	0	1	1	1	6	Fair
#1109	Khalili et al.	2017	1	0	1	1	0	1	1	1	6	Fair
#1128	Spaite et al.	2017	1	0	1	1	0	1	1	1	6	Fair
#1183	Aiolfi et al	2017	1	0	1	1	0	1	1	1	6	Fair
#1204	Spaite et al.	2017	1	0	1	1	0	1	1	1	6	Fair
#1222	Spaite et al.	2017	1	0	1	1	0	1	1	1	6	Fair
#1345	Hasanin et al.	2016	1	0	1	1	0	1	1	1	6	Fair
#1474	Herrera-Melero et al.	2015	1	0	1	1	0	1	1	1	6	Fair
#1561	Newgard et al.	2015	1	1	1	1	1	1	1	1	8	Good
#1726	Tohme et al.	2014	1	0	1	1	0	1	1	1	6	Fair
#1741	Fuller et al.	2014	1	0	1	1	0	1	1	1	6	Fair
#1887	Muehlschlegel et al.	2013	1	0	1	1	0	1	1	1	6	Fair
#1917	Jacobs et al.	2013	1	0	1	1	0	1	1	0	5	Fair
#1971	Farahvar et al	2012	1	1	1	1	2	1	1	1	9	Good
#2193	Zafar et al.	2011	1	1	1	0	2	1	1	1	8	Good
#2201	Franschman et al.	2011	1	1	1	1	1	1	1	1	8	Good
#2223	Farahvar et al.	2011	1	1	1	1	1	1	1	1	8	Good
#2262	Vos et al.	2010	1	1	1	1	2	1	1	1	9	Good
#2337	Petroni et al.	2010	1	1	1	1	2	1	1	1	9	Good
#2457	Utomo et al.	2009	1	1	1	1	0	1	1	1	7	Good
#2486	Chamoun et al.	2009	1	1	1	1	2	1	1	1	9	Good
#2563	DuBose et al.	2008	1	1	1	1	2	1	1	1	9	Good
#2599	Steyerberg et al.	2008	1	1	1	1	2	1	1	1	9	Good
#2606	Hartl et al.	2008	1	1	1	1	2	0	0	0	6	Fair
#2606	Chi et al.	2008	0	0	1	1	1	1	1	1	6	Fair

#2961	Shafi et al.	2005	1	1	1	1	2	1	1	1	9	Good
#2994	Hukkelhoven et al.	2005	1	1	1	1	2	1	1	1	9	Good
#3449	Manley et al.	2001	1	1	1	1	2	1	1	1	9	Good
#4974	Lenstra et al	2020	1	0	1	1	0	1	1	1	6	Fair
#5031	Para et al.	2018	1	0	1	1	0	1	1	1	6	Fair
#5108	Krishnamoorthy et al.	2015	1	0	1	1	0	1	1	1	6	Fair
#9550	Yeung et al.	2021	1	0	1	1	0	1	1	1	6	Fair
#13506	Asmar et al.	2021	1	1	1	1	1	1	1	1	8	Good
#14404	Pin-On et al.	2017	1	0	1	1	0	1	1	1	6	Fair
#20083	Song et al.	2023	1	0	1	1	0	1	1	1	6	Fair
#15888	Farahvar et al.	2011	1	1	1	1	1	1	1	1	8	Good

Column header legend: 1= Representativeness of the Exposed Cohort, 2 = Selection of the Non-Exposed Cohort, 3 = Ascertainment of Exposure, 4 = Demonstration that Outcome of Interest Was Not Present at Start of Study, 5 = Comparability of Cases and Controls on the Basis of the Design or Analysis, 6 = Assessment of Outcome, 7 = Was Follow-Up Long Enough for Outcomes to Occur, 8 = Adequacy of Follow Up of Cohorts, 9 = Total Score.

© 2024 Lee JW et al. *JAMA Network Open.*

eTable 6: Heterogeneity analysis for continuous variables

Variable	I2	R2	p-value
1. Sample size	91·48	7·62	0·07
2. Male %	90·58	7·34	0·03
3. BP cutoff	89·37	26·53	0·001
4. Mean age	92·02	0	0·43

© 2024 Lee JW et al. *JAMA Network Open.*

eTable 7: Heterogeneity analysis for categorical variables

Group	tau2	I2
Sample size category		
1001-5000	0·121	83·24
200-1000	0·529	85·20
<200	0·000	0
>5000	0·158	94·05
BP category		
90	0·244	92·04
<90	0·000	0
>90	0·046	82·20
TBI severity		
Both	0·363	88·47
Moderate	0·000	0
Severe	0·181	92·52
TBI scale		
AIS	0·151	92·01
GCS	0·278	91·20
Hypoxia adjusted		
No	0·318	95·82
Yes	0·053	71·47
Location		
ALL	0·270	88·54
ED	0·273	92·61
EMS	0·065	81·52
ICU	0·451	65·70
Trauma type		
Isolated	0·495	97·10
Polytrauma	0·131	89·01
Age category		
40-50	0·131	84·19
50-60	0·175	94·34
<40	0·289	91·77
>60	0·016	26·60
Combined 1-3	0·186	92·21

© 2024 Lee JW et al. *JAMA Network Open.*

eTable 8: Leave one out analysis for studies reporting adjusted OR (60 studies)

Exp(theta) is the overall adjusted OR.

Omitted study	exp(theta)	[95% conf. interval]	p-value
Aiolfi 2017	2·216	1·952-2·516	0·001
Aiolfi 2018	2·146	1·914-2·407	0·001
Czorlich 2017	2·219	1·956-2·519	0·001
DuBose 2008	2·241	1·977-2·540	0·001
Farahvar 2011	2·218	1·954 -2·516	0·001
Farahvar 2012	2·221	1·957-2·521	0·001
Franschman 2011	2·206	1·946-2·499	0·001
Fuller 2014 (a)	2·199	1·943-2·490	0·001
Fuller 2014 (b)	2·209	1·950-2·503	0·001
Fuller 2014 (c)	2·212	1·951-2·507	0·001
Fuller 2014 (d)	2·221	1·958-2·519	0·001
Fuller 2014 (e)	2·225	1·961-2·524	0·001
Huang 2022 (a)	2·212	1·953-2·505	0·001
Huang 2022 (b)	2·228	1·967-2·524	0·001
Hukkelhoven 2005	2·219	1·954-2·519	0·001
Jacobs 2013	2·210	1·950-2·506	0·001
Kamal 2016	2·175	1·927-2·456	0·001
Kamal 2021	2·158	1·919-2·426	0·001
Khalili 2017	2·164	1·922-2·436	0·001
Krishnamoorthy 2015	2·230	1·965-2·531	0·001
Lenstra 2020	2·208	1·949-2·501	0·001
Merck 2019	2·238	1·974-2·536	0·001
Newgard 2015 (a)	2·228	1·964-2·527	0·001
Newgard 2015 (b)	2·248	1·989-2·541	0·001
Newgard 2015 (c)	2·253	1·997-2·542	0·001
Petroni 2010	2·207	1·949-2·499	0·001
Rice 2023 (a)	2·227	1·962-2·528	0·001
Rice 2023 (b)	2·211	1·949-2·509	0·001
Rice 2023 (c)	2·190	1·934-2·479	0·001
Schellenberg 2021	2·210	1·949-2·505	0·001
Shibahashi 2017	2·215	1·952-2·513	0·001
Shibahashi 2018 (a)	2·234	1·969-2·535	0·001
Shibahashi 2018 (b)	2·238	1·974-2·537	0·001
Shibahashi 2018 (c)	2·235	1·970-2·535	0·001
Shibahashi 2018 (d)	2·222	1·957-2·522	0·001
Shibahashi 2018 (e)	2·209	1·946-2·507	0·001
Shibahashi 2018 (f)	2·205	1·944-2·502	0·001
Shibahashi 2021 (a)	2·236	1·971-2·536	0·001
Shibahashi 2021 (b)	2·245	1·983-2·542	0·001
Shibahashi 2021 (c)	2·233	1·968-2·534	0·001
Shibahashi 2021 (d)	2·225	1·960-2·526	0·001
Shibahashi 2021 (e)	2·212	1·949-2·511	0·001
Shibahashi 2021 (f)	2·210	1·947-2·508	0·001
Spaite 2017 (1128)	2·243	1·980-2·541	0·001
Spaite 2017 (1222)	2·213	1·950-2·512	0·001
Spaite 2017 (a)	2·198	1·940-2·489	0·001
Spaite 2017 (b)	2·207	1·946-2·503	0·001
Spaite 2017 (c)	2·217	1·953-2·516	0·001
Spaite 2017 (d)	2·211	1·949-2·510	0·001
Spaite 2017 (e)	2·211	1·949-2·509	0·001
Spaite 2017 (f)	2·223	1·958-2·523	0·001
Spaite 2017 (g)	2·223	1·958-2·523	0·001
Spaite 2017 (h)	2·221	1·956-2·521	0·001
Spaite 2017 (i)	2·223	1·958-2·524	0·001
Spaite 2017 (j)	2·225	1·960-2·526	0·001
Spaite 2017 (k)	2·230	1·965-2·531	0·001
Spaite 2017 (l)	2·233	1·968-2·533	0·001
Spaite 2017 (m)	2·233	1·968-2·533	0·001
Tohme 2014	2·219	1·956-2·517	0·001
Zafar 2011	2·209	1·947-2·507	0·001
exp(theta)	2·217	1·958-2·510	0·001

Fuller 2014 sub-studies include different hypotension thresholds of a) 70mmHg, b) 80mmHg, c) 90 mmHg, d)100mmHg, e)110mmHg. Huang 2022 sub-studies include a) moderate TBI) and b) severe TBI. Newgard 2015 sub-studies include different hypotension thresholds of a) <90mmHg, b) 90-105mmHg, c) 105-120mmHg. Rice 2023 sub-studies include blood pressure measurement in a) EMS, b) ED, c) EMS and ED. Shibahashi 2018 and 2021 sub-studies include different blood pressure thresholds of a) 100-109mmHg, b) 90-99mmHg, c) 80-89mmHg, d) 70-79mmHg, e) <70mmHg, f) <110mmHg. Spaite 2017 sub-studies include blood pressure thresholds increments of 5mmHg from a) <60mmHg to m) <120mmHg.

© 2024 Lee JW et al. *JAMA Network Open.*

eTable 9: Leave one out analysis for studies reporting crude OR (50 studies)

Omitted study	Crude HR	[95% conf. interval]	p-value
Chamoun 2009	3·888	3·082-4·906	0·001
Corral 2012	3·812	3·013-4·822	0·001
Franschman 2011	3·828	3·026-4·842	0·001
Fuller 2014 (a)	3·688	2·947-4·616	0·001
Fuller 2014 (b)	3·741	2·967-4·716	0·001
Fuller 2014 (c)	3·805	3·007-4·815	0·001
Fuller 2014 (d)	3·845	3·039-4·865	0·001
Fuller 2014 (e)	3·884	3·076-4·906	0·001
Heppekcan 2019	3·761	2·982-4·744	0·001
Herrera-Melero 2015	3·865	3·057-4·886	0·001
Huang 2022 (a)	3·817	3·026- 4·814	0·001
Huang 2022 (b)	3·886	3·083- 4·897	0·001
Kamal 2016	3·721	2·957- 4·681	0·001
Kamal 2021	3·705	2·951- 4·651	0·001
Kim 2018 (a)	3·845	3·043- 4·859	0·001
Kim 2018 (b)	3·858	3·054- 4·872	0·001
Krishnamoorthy 2015	3·810	3·010-4·822	0·001
Lenstra 2020	3·849	3·045-4·865	0·001
Manley 2001	3·829	3·029-4·840	0·001
Muehlschlegel 2013	3·819	3·019-4·832	0·001
Newgard 2015 (a)	3·869	3·061-4·889	0·001
Newgard 2015 (b)	3·946	3·144-4·953	0·001
Newgard 2015 (c)	3·972	3·175-4·969	0·001
Para 2018	3·777	2·993-4·766	0·001
Petroni 2010	3·780	2·995-4·771	0·001
Pin-On 2017 (a)	3·840	3·041-4·848	0·001
Pin-On 2017 (b)	3·847	3·049-4·855	0·001
Pin-On 2017 (c)	3·888	3·086-4·898	0·001
Pin-On 2017 (d)	3·780	2·997-4·767	0·001
Rauch 2021	3·863	3·056-4·882	0·001
Rice 2023 (BOTH)	3·707	2·952-4·656	0·001
Rice 2023 (ED)	3·747	2·969-4·729	0·001
Rice 2023 (EMS)	3·813	3·012-4·828	0·001
Shafi 2005 (ED)	3·758	2·975-4·748	0·001
Shafi 2005 (EMS)	3·715	2·955-4·670	0·001
Shibahashi 2021 (a)	3·923	3·116-4·939	0·001
Shibahashi 2021 (b)	3·786	2·993-4·789	0·001
Shibahashi 2021 (c)	3·816	3·014-4·831	0·001
Shibahashi 2021 (d)	3·846	3·040-4·868	0·001
Shibahashi 2021 (e)	3·885	3·076-4·907	0·001
Shibahashi 2021 (overall)	3·887	3·077-4·909	0·001
Shibahasi 2017	3·864	3·056-4·885	0·001
Song 2023 (a)	3·891	3·082-4·912	0·001
Spaite 2017 (1128)	3·779	2·988-4·779	0·001
Spaite 2017 (1222)	3·808	3·008-4·820	0·001
Spaite 2022	3·788	2·994-4·793	0·001
Tohme 2014	3·849	3·044-4·867	0·001
Vos 2010	3·889	3·086-4·900	0·001
Yeung 2011	3·715	2·955-4·670	0·001
Zeiler 2021	3·857	3·054-4·871	0·001
exp(theta)	3·822	3·036-4·813	0·001

Fuller 2014 sub-studies include different hypotension thresholds of a) 70mmHg, b) 80mmHg, c) 90 mmHg, d)100mmHg. Huang 2022 sub-studies include a) moderate TBI) and b) severe TBI. Kim 2018 sub-studies include blood pressure thresholds of a) <90mmHg and b) <110 mmHg. Newgard 2015 sub-studies include different hypotension thresholds of a) <90mmHg, b) 90-105mmHg, c) 105-120mmHg. Pin-On 2017 sub-studies include a) severe TBI, blood pressure <20% baseline measured during induction, b) severe TBI, blood pressure <20% baseline measured intra-operatively, c) moderate TBI, blood pressure <90mmHg measured during induction, d) moderate TBI, blood pressure <90mmHg measured intra-operatively. Shibahashi 2021 sub-studies include different blood pressure thresholds of a) 100-109mmHg, b) 90-99mmHg, c) 80-89mmHg, d) 70-79mmHg, e) <70mmHg.

© 2024 Lee JW et al. *JAMA Network Open.*

eTable 10: Heterogeneity analysis for sub-studies using multi-level meta-analysis

Assumption	Effect estimate (95% CI)	Heterogeneity (%)
No dependence	2.22 (1.96-2.51)	92
Dependence (multiplicative model)	2.60 (2.01-3.35)	77
No sub-studies (sensitivity)	2.96 (2.22-3.94)	92

© 2024 Lee JW et al. *JAMA Network Open.*

eFigure 1: Forest plot showing crude association of hypotension on adverse outcome (vegetative state/mortality) in patients with moderate to severe traumatic brain injury (Effect estimate=unadjusted odds ratio) (50 studies)

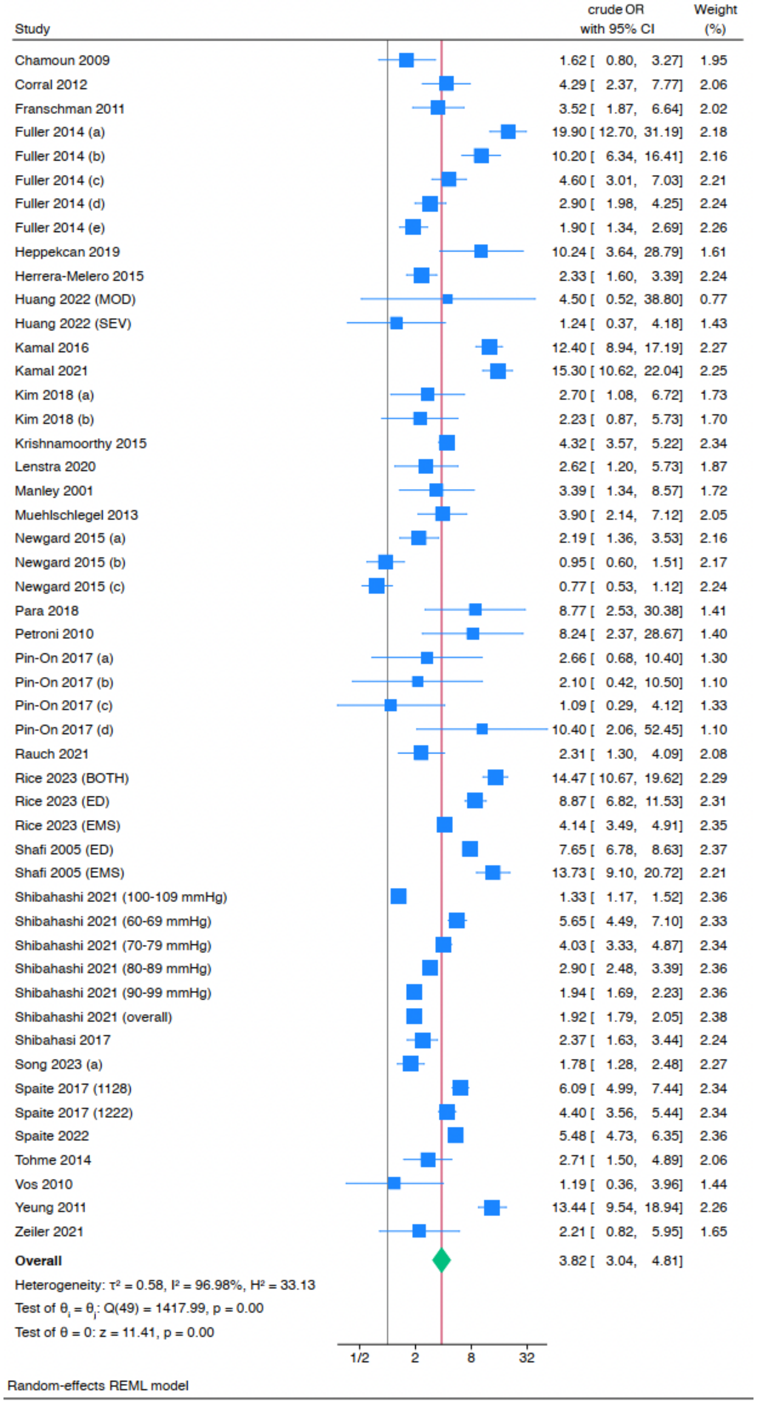

Legend: Fuller 2014 sub-studies include different hypotension thresholds of a) 70mmHg, b) 80mmHg, c) 90 mmHg, d)100mmHg e)110mmHg. Kim 2018 sub-studies include blood pressure thresholds of a) <90mmHg and b) <110 mmHg. Newgard 2015 sub-studies include different hypotension thresholds of a) <90mmHg, b) 90-105mmHg, c) 105-120mmHg. Pin-On 2017 sub-studies include a) severe TBI, blood pressure <20% baseline measured during induction, b) severe TBI, blood pressure <20% baseline measured intra-operatively, c) moderate TBI, blood pressure <90mmHg measured during induction, d) moderate TBI, blood pressure <90mmHg measured intra-operatively.

© 2024 Lee JW et al. *JAMA Network Open.*

eFigure 2: Forest plot showing incidence of hypotension by subgroups in patients with moderate to severe traumatic brain injury (44 studies)

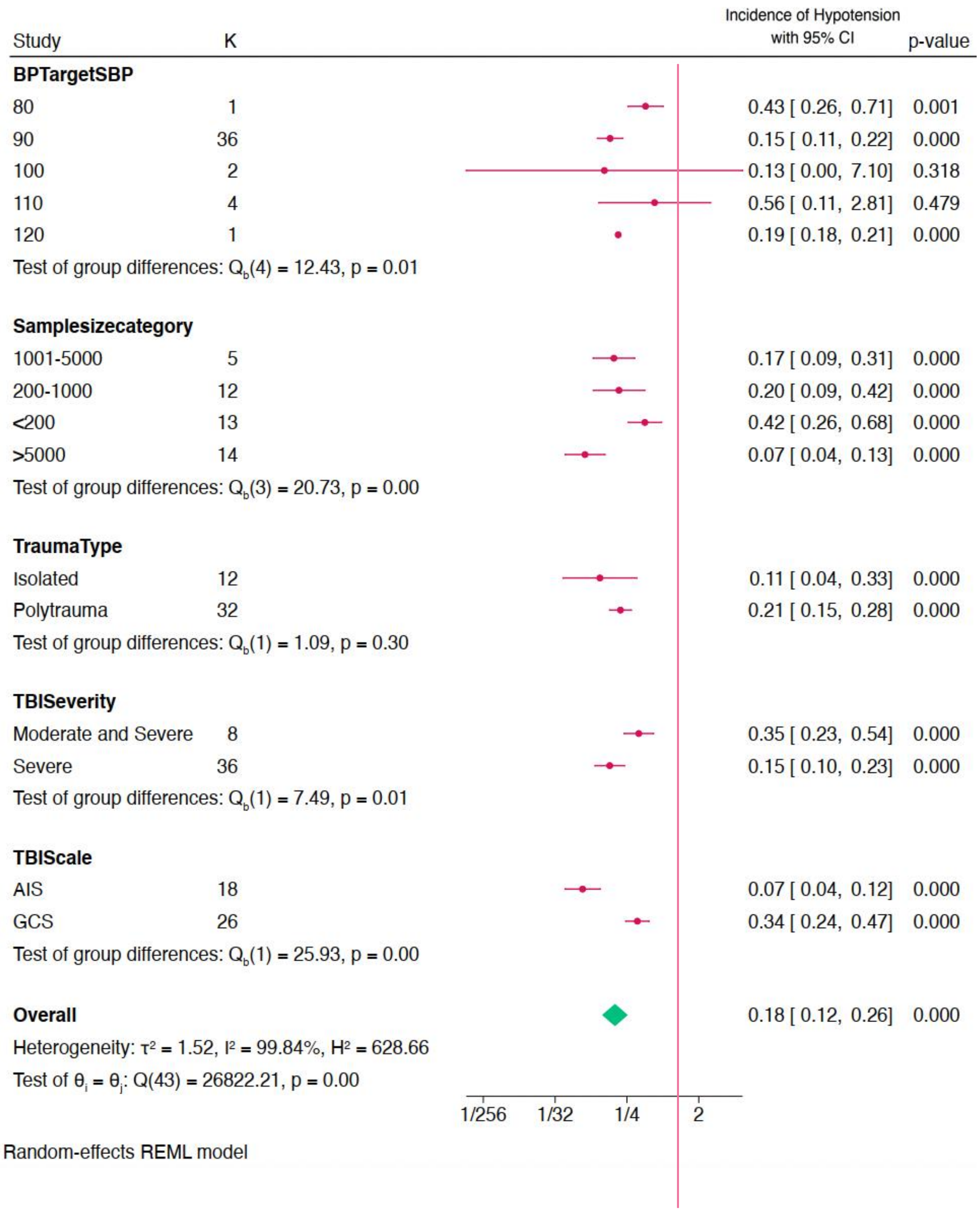

Legend: K indicates the number of studies. Vertical red line indicates line of no effect. Green diamond indicates an overall pooled effect. Horizontal red lines indicate 95% confidence interval of respective study.

© 2024 Lee JW et al. *JAMA Network Open.*

eFigure 3: Forest plot showing association of hypotension on adverse outcome (vegetative state/mortality) in patients with moderate to severe traumatic brain injury (effect estimate=adjusted odds ratio) (67 studies with or without complete data on sample size)

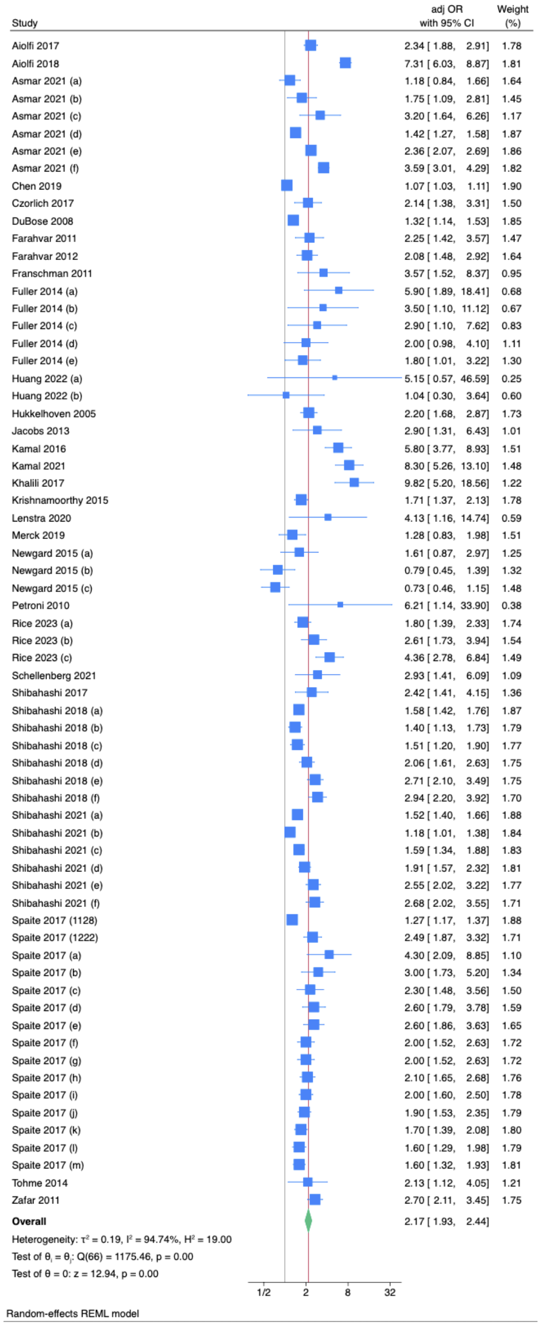

Legend: Asmar 2021 sub-studies include a) moderate TBI and blood pressure 90-109mmHg, b) moderate TBI and blood pressure 70-90mmHg, c) moderate TBI and blood pressure <70mmHg, d) severe TBI and blood pressure 90-109mmHg, e) severe TBI and blood pressure 70-90mmHg, f) severe TBI and blood pressure <70mmHg. Fuller 2014 sub-studies include different hypotension thresholds of a) 70mmHg, b) 80mmHg, c) 90 mmHg, d)100mmHg e)110mmHg. Huang 2022 sub-studies include a) moderate TBI) and b) severe TBI. Newgard 2015 sub-studies include different hypotension thresholds of a) <90mmHg, b) 90-105mmHg, c) 105-120mmHg. Rice 2023 sub-studies include blood pressure measurement in a) EMS, b) ED, c) EMS and ED. Shibahashi 2018 and 2021 sub-studies include different blood pressure thresholds of a) <110mmHg, b) 100-109mmHg, c) 90-99mmHg, d) 80-89mmHg e) 70-79mmHg, f) <70mmHg. Spaite 2017 sub-studies include blood pressure thresholds increments of 5mmHg from a) <60mmHg to m) <120mmHg.

© 2024 Lee JW et al. *JAMA Network Open.*

eFigure 4: Funnel plot (contour-enhanced) for publication bias.

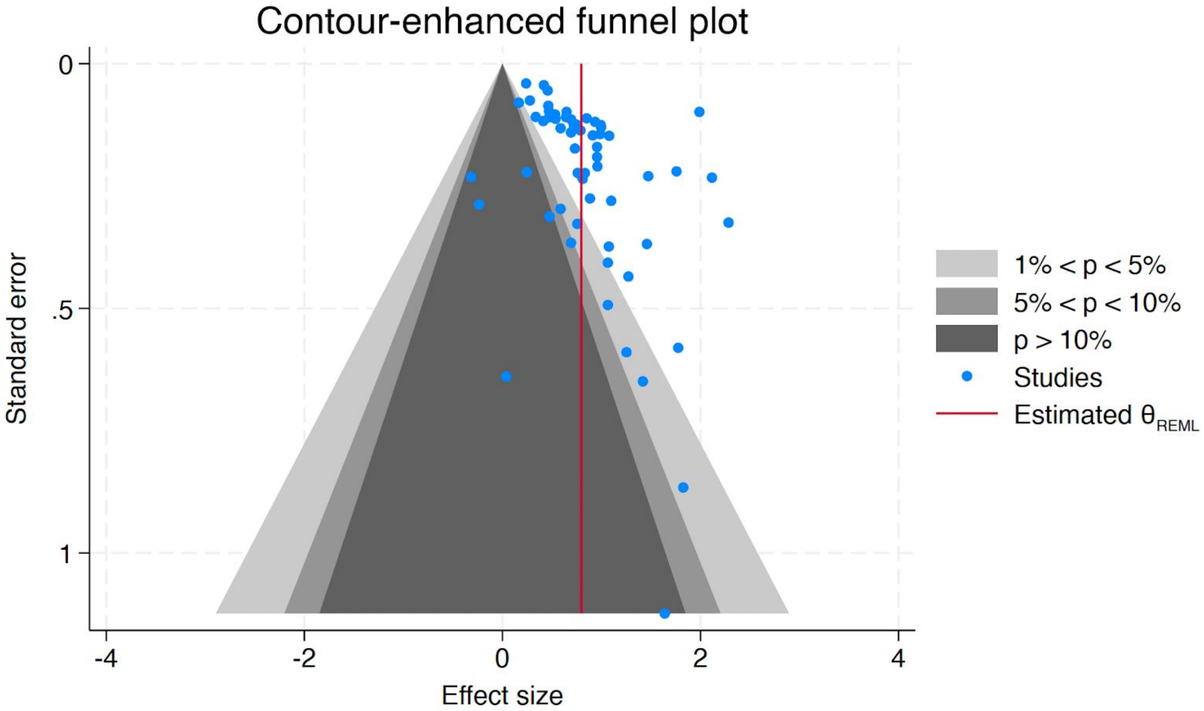

Legend: Blue dots represent individual studies. Shades of grey represent different p values. Red line indicates estimated theta random effects model.

© 2024 Lee JW et al. *JAMA Network Open.*

eFigure 5: Galbraith Plot for exhibiting outliers.

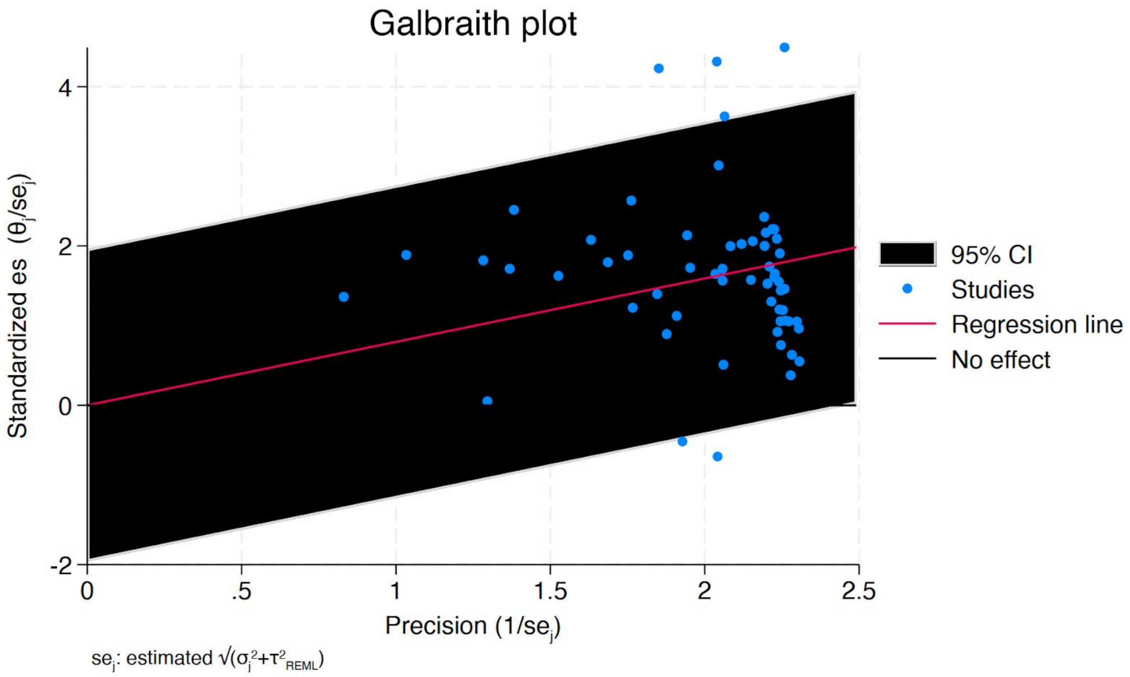

Legend: Blue dots represent individual studies. Black indicates 95% CI. Red line indicates regression line.

© 2024 Lee JW et al. *JAMA Network Open.*

eFigure 6: Trim fill funnel plot for publication bias.

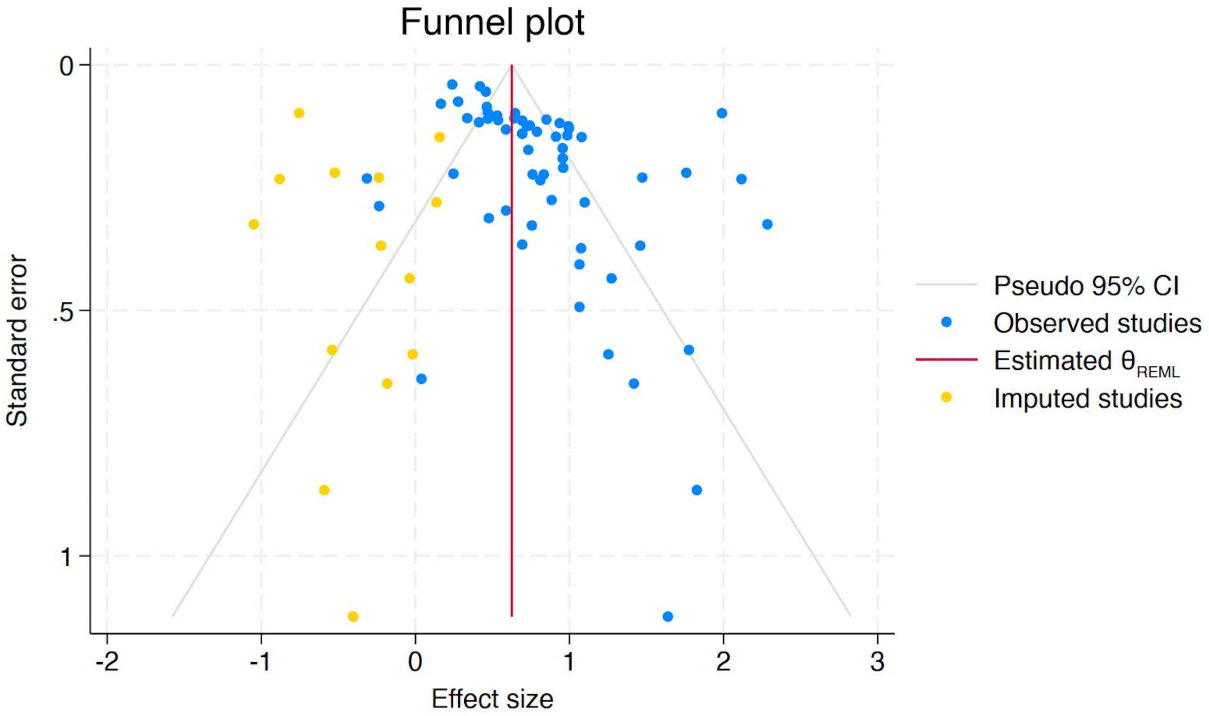

Legend: Blue dots represent observed studies. Yellow dots represent imputed studies. White lines represent pseudo 95% confidence intervals. Red line represents estimated theta random effects model.

© 2024 Lee JW et al. *JAMA Network Open.*
