# Supplementary material for: Hypotension and Adverse Outcomes in Moderate to Severe Traumatic Brain Injury: A Systematic Review and Meta-Analysis
Source: JAMA Netw Open. 2024 Nov 11;7(11):e2444465. doi: 10.1001/jamanetworkopen.2024.44465 (PMC11555550; doi:10.1001/jamanetworkopen.2024.44465)
Supplement: Supplement 2. — Data Sharing Statement [file jamanetwopen-e2444465-s002.pdf]

Data Sharing Statement

Lee. Hypotension and Adverse Outcomes in Moderate to Severe Traumatic Brain Injury. *JAMA Netw Open*. Published November 11, 2024. doi:10.1001/jamanetworkopen.2024.44465

Data
Data available: Yes
Data types: Other (please specify)
Additional Information: Data extracted from articles and analysis plans
How to access data: Requests can be made to tumul.chowdhury@uhn.ca
When available: With publication

Supporting Documents
Document types: None

Additional Information
Who can access the data: Data extracted from articles and analysis plans will be made available on request for the use of umbrella reviews. Requests can be made to tumul.chowdhury@uhn.ca
Types of analyses: Umbrella Reviews
Mechanisms of data availability: With investigator support, signed data access agreement
